# Open-label, phase I, pharmacokinetic studies of abiraterone acetate in healthy men

**DOI:** 10.1007/s00280-012-1865-3

**Published:** 2012-04-18

**Authors:** M. Acharya, A. Bernard, M. Gonzalez, J. Jiao, R. De Vries, N. Tran

**Affiliations:** 1 Janssen Research and Development, LLC, 920 Route 202 South, Raritan, NJ 08869 USA; 2 Janssen Research and Development, LLC, Beerse, Belgium

**Keywords:** Abiraterone acetate, Dose-proportionality, Pharmacodynamics, Pharmacokinetics, Prostate cancer

## Abstract

**Purpose:**

To evaluate pharmacokinetics, safety, and tolerability of abiraterone acetate (AA) in healthy men.

**Methods:**

Two phase I studies (dose-escalation study and dose-proportionality study) were conducted in healthy men aged 18–55 years. All subjects received 4 consecutive single doses of AA (250, 500, 750 and 1,000 mg). The dose-escalation study subjects (*N* = 33) received AA doses in a sequential manner, starting with the lowest dose. The dose-proportionality study subjects (*N* = 32) were randomly allocated (1:1:1:1) to receive each of the 4 doses in a four-way crossover design.

**Results:**

A dose-related increase in abiraterone exposure was observed in both studies. Over the evaluated dose range, the mean abiraterone maximum plasma concentrations increased from 26 to 112 ng/mL in dose-escalation study and from 40 to 125 ng/mL in dose-proportionality study; the mean area under the plasma concentration–time curve from 0 to the last measurable plasma concentration increased from 155 to 610 ng.h/mL in dose-escalation study, and from 195 to 607 ng.h/mL in dose-proportionality study. In the dose-proportionality study, abiraterone exposure was dose proportional between 1,000 and 750 mg doses; however, the exposure was slightly greater than dose proportional when exposures at 500 and 250 mg doses were compared with the exposure at 1,000 mg. Single doses of AA were well tolerated in healthy men, and safety profile was consistent with its known toxicities in CRPC patients.

**Conclusion:**

Systemic exposure to abiraterone increased with increasing doses of AA (250–1,000 mg) in healthy men; AA was well tolerated in this population.

## Introduction

Prostate cancer is the second leading cause of cancer deaths, accounting for 9 % of all cancer-related death in men [1]. Androgen deprivation therapy (ADT) is a standard treatment for advanced, adjuvant, and non-adjuvant stages of the disease and involves surgical (orchiectomy) or medical (luteinizing hormone (LH)-releasing hormone agonists, or antiandrogens) intervention [2, 3]. Although ADT induces a remission in 80–90 % of patients with advanced disease [4], majority of patients eventually develop castration-resistant prostate cancer (CRPC), an androgen-independent phenotype and the lethal form of the disease, in which <20 % of patients survive beyond 3 years [5]. New therapeutic strategies, which can offer better protection against CRPC, would be useful.

The enzyme 17α-hydroxylase/C17, 20-lyase (CYP17) catalyzes critical reactions in the synthesis of androgens [6]. Ketoconazole, the non-selective inhibitor of CYP17 enzyme, presents modest antitumor activity and is associated with an increase in androgenic steroids at disease progression, indicating incomplete target blockade [7]. Abiraterone is a selective and irreversible inhibitor of CYP17 enzyme and is 10–30-fold more potent than ketoconazole [8]. A prodrug of abiraterone, abiraterone acetate (AA), was developed to overcome its poor bioavailability. Abiraterone acetate (ZYTIGA^**®**^ **)** in combination with prednisone is approved in the United States [9], Canada [10], and Europe [11] for the treatment of male subjects with metastatic castration-resistant prostate cancer, who have received prior chemotherapy containing docetaxel.

The safety and tolerability of AA was demonstrated in two dose-escalation studies in CRPC patients, and no dose-limiting toxicity was identified at doses up to 2,000 mg [5, 12]. In this report, we present the results from two phase I studies (dose-escalation study and dose-proportionality study) in healthy men, which evaluated pharmacokinetics (PK) and safety of AA at doses of 250, 500, 750, and 1,000 mg. Additionally, in the dose-proportionality study, pharmacodynamic evaluation was carried out to assess the effect of abiraterone on levels of testosterone and LH.

## Methods

### Study population

Healthy men between 18 and 55 years of age (inclusive), with body mass index within 18 to 32 kg/m^2^ (inclusive), were enrolled in both studies. Subjects were in good general health as determined by medical history, physical examination, vital signs, 12-lead electrocardiogram (ECG), and clinical laboratory measurements; they had negative test results for hepatitis B surface antigen, hepatitis C virus antibody, human immunodeficiency virus antibody, selected drugs of abuse, and breathalyzer alcohol and had not consumed nicotine or nicotine-containing products in 1 year before enrollment. All subjects used an acceptable method of contraception from day-1 until 30 days following the completion of studies.

Exclusion criteria were a significant history or clinical manifestation of any significant diseases or disorders, history of stomach or intestinal surgery or resection that would potentially alter absorption or excretion of drugs, abnormal ECG, or hypersensitivity reaction to the study drug or related compounds.

Subjects agreed to refrain from consuming grapefruit- or caffeine-containing products (for 72 h before day-1 until after collection of 96-h PK sample for the dose-escalation study and throughout the study for the dose-proportionality study) and alcohol-containing products (for 24 h before day-1 until the end of study in both studies). Additionally, they refrained from strenuous exercise from 48-h before day-1 and during the period of confinement at the clinical research unit (CRU) for the dose-escalation study and throughout the study for the dose-proportionality study.

Concomitant medications were not allowed during both studies with the exception of acetaminophen and non-steroidal anti-inflammatory drugs, which were allowed after the 96-h PK sample collection.

Both studies were conducted in accordance with the ethical principles that have their origin in the declaration of Helsinki and that are consistent with good clinical practices, applicable regulatory requirements, and in compliance with the respective protocols. The study protocols were reviewed and approved by an independent institutional review board (Florida, USA). All subjects provided written informed consent before entering the studies.

### Study designs

The dose-escalation study, conducted from December 17, 2008, to April 02, 2009, was an open-label study with a 14-day screening phase and up to 88-day treatment phase (approximately 22 days for each of the 4 single-dose cohorts). The dose-proportionality study, conducted from March 30, 2010, to May 1, 2010, was an open-label, randomized, four-way crossover study, with a 21-day screening phase and a 28-day treatment phase (consisting of 4 consecutive, 7-day treatment periods). Both studies were conducted at Covance Clinical Research Unit Inc., Texas, United States of America.

Abiraterone acetate (manufactured by Pharmaceutical International, Inc., Maryland) was provided as 250-mg tablets. In both studies, AA was administered at doses of 250, 500, 750, and 1,000 mg/day. In the dose-escalation study, these doses were administered to subjects in 4 dose cohorts in a sequential manner, starting with the lowest dose (250 mg/day). Dose administration for subsequent cohorts was started at least 15 days after dosing of the preceding cohort and after the safety data were reviewed and assessed as not having any safety concerns such as clinically significant adverse event indicative of drug intolerance; clinically significant finding for physical examination, 12-lead ECG, or vital signs; or clinically significant change in laboratory parameter. Due to an error in PK sample processing at the CRU for the Cohort 2 dose level (500 mg), another 500-mg dose level cohort was added and designated as Cohort 2a. The bio-analytical data for Cohort 2 was not evaluated.

In the dose-proportionality study, 32 eligible subjects were randomized (1:1:1:1) to receive 1 of the 4 treatment sequences: ADBC, BACD, CBDA, DCAB. Each treatment sequence included 4 doses of AA: 250 mg/day (treatment A), 500 mg/day (treatment B), 750 mg/day (treatment C), and 1,000 mg/day (treatment D). For each subject, dose administration for subsequent treatment in the treatment sequence was started 7 days after dosing of the preceding treatment.

All subjects fasted for at least 10 h before and 4 h after AA administration in both studies. Subjects were confined to CRU one day before every administration of AA and until collection of 24 h PK sample the following day. In addition, subjects visited CRU on days 3, 4, 5, 8, and 15 of each dose cohort in dose-escalation study and on days 3, 4, and 7 of each treatment period in the dose-proportionality study, for study procedures.

### Pharmacokinetic evaluation

During both studies, blood samples were collected for the measurement of AA and abiraterone concentrations in plasma; samples were collected from the peripheral vein (via an indwelling catheter or direct venipuncture), within 0.5 h before the dose (predose sample) and at 0.25, 0.5, 1, 1.5, 2, 3, 4, 6, 8, 12, 24, 48, 72, and 96 h postdose. Additionally, in the dose-proportionality study, blood samples for evaluation of serum testosterone and LH were also collected on day 1 (predose and 2 and 12 h postdose), 2, 3, 4, and 5 of each cohort and at the end of study or early withdrawal visit.

### Bioanalytical method

Concentrations of abiraterone and AA were determined using a validated bioanalytical method employing ultra performance liquid chromatography with tandem mass spectrometric detection. The calibration range for abiraterone was 0.2–200 ng/mL for the dose-escalation study and 0.2–500 ng/mL for the dose-proportionality study. The calibration range for AA was 0.2–50 ng/mL for both studies.

### Data analysis

For the dose-escalation study, non-compartmental analysis was performed using WinNonlin (Pharsight Corporation, Version 5.2). SAS version 9.1 was used for all statistical calculations, tables, and plots. For the dose-proportionality study, non-compartmental analysis was performed using WinNonlin (Version 4.0.1b). SAS version 9.1 was used for confidence interval testing. Tables and plots were created using PKAA 1.0, which utilized Microsoft Excel 2000.

### Pharmacokinetic parameters

Pharmacokinetic parameters calculated from plasma concentration–time data were maximum plasma concentration (*C*_max_), time to reach *C*_max_ (*t*_max_), and area under the plasma concentration–time curve (AUC) from 0 to the last measurable plasma concentration as calculated by the linear trapezoidal rule (AUC_0–last_); AUC extrapolated to infinity (AUC_0–∞_), calculated as: AUC_0–∞_ = AUC_0–last_ + C_t_/λ_z_, where C_t_ was the last measurable plasma concentration and λ_z_ was the apparent terminal phase rate constant calculated as the magnitude of the slope of the linear regression of the log concentration versus time profile during the terminal phase; and apparent plasma terminal elimination half-life *t*_1/2_ (whenever possible), where *t*_1/2_ = (ln_2_)/λ_z_.

### Safety evaluations

Safety assessments included monitoring treatment-emergent adverse events (TEAEs), clinical laboratory tests, ECG, vital sign measurements, and physical examinations. The severity of adverse events (AEs) was assessed using the National Cancer Institute Common Terminology Criteria for AEs (NCI CTCAE, version 3.0, grades 1, 2, 3, and 4) [13]. In addition, total testosterone and LH levels on days-1, 3, 5, 8, and 15 of each cohort in the dose-escalation study and on day 1 (predose and 2 and 12 h postdose) and day 2 through day 5 in each treatment period in the dose-proportionality study were evaluated. These evaluations were repeated approximately 7 days after the end of study, for subjects whose serum testosterone levels were not within the normal limits or below the baseline value at the end of study. In the dose-proportionality study, testosterone levels were monitored every 7 days until the levels returned to baseline or to within normal limits.

### Statistical analysis

In both studies, PK analysis sets included all subjects who received the study drug and had at least 1 valid PK parameter estimate, and the safety analysis sets included all subjects who received at least one dose of study drug.

Plasma concentration data of abiraterone and all estimated PK parameters were summarized descriptively for both studies. For the dose-proportionality study, least squares means (LSM) and intra-subject variance were estimated using a mixed-effect model, which included treatment, period, and treatment sequence as fixed effects and participant as a random effect.

### Sample size determination

Approximately 32 subjects were to be enrolled in the dose-escalation study based upon samples sizes used in previous PK studies of similar nature. For the dose-proportionality study, the sample size of 24 subjects (using an estimated intra-subject coefficient of variation of 21 % for AUC_0–last_ and AUC_0–∞_) was sufficient for the point estimates of the ratio of AUC_0–last_ and AUC_0–∞_ to fall within 90–111 % of the true value with 90 % confidence. This sample size was also sufficient for the point estimate of the ratio of *C*_max_ to fall within 86–116 % of the true value with 90 % confidence using an estimated intra-participant coefficient of variation of 31 % for *C*_max_. Thirty-two subjects were randomized in the dose-proportionality study in order to obtain at least 24 subjects who completed the study.

## Results

### Subject disposition and baseline characteristics

In the dose-escalation study, 40 men were screened, 33 were enrolled, 8 were assigned to each dose cohort, and all completed the study. Dose cohort 2a (500 mg/day, repetition of cohort 2) included 7 subjects from cohort 2 and one new participant. In the dose-proportionality study, 32 men were enrolled, 27 completed the study, 3 discontinued due to non-compliance, and 2 were lost to follow-up. Baseline and demographic characteristics of subjects from both studies are presented in Table 1.

**Table 1:** Demographic and baseline characteristics of subjects (safety analysis set)

	Dose-escalation study (*N* = 33)	Dose-proportionality study (*N* = 32)
Age (years), mean (range)	37 (22–54)	35.5 (22–54)
*Ethnicity, n (%)*		
Hispanic/latino	11 (33.3)	12 (37.5)
Non-hispanic/latino	22 (66.7)	20 (62.5)
*Race, n (%)*		
White	19 (57.6)	22 (68.8)
Black or African American	13 (39.4)	7 (21.9)
Asian	1 (3)	1 (3.1)
American Indian/Alaska Native	0	1 (3.1)
Other	0	1 (3.1)
BMI (kg/m^2^), mean (range)	26.0 (19.2–32.0)	26.6 (21–30)

*BMI* body mass index

### Pharmacokinetics of abiraterone acetate

The PK analysis was not performed for AA as the majority of plasma concentrations for AA were below the limit of quantification in both studies.

### Pharmacokinetics of abiraterone

**Fig. 1:**
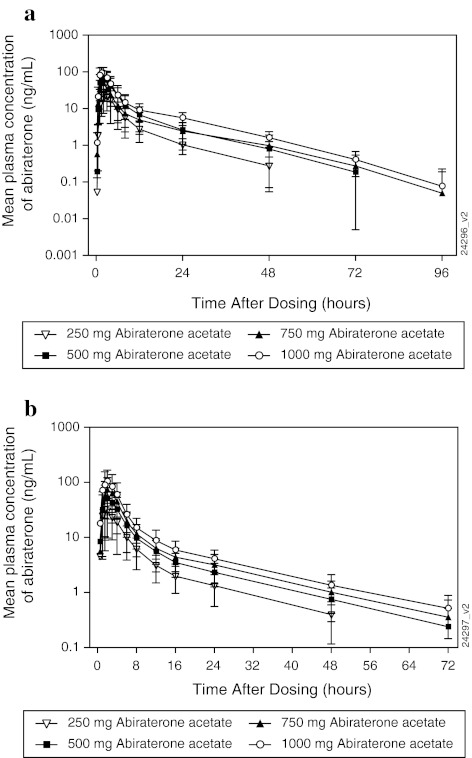
**a** Mean (SD) plasma concentration–time profile of abiraterone in dose-escalation study. **b** Mean (SD) plasma concentration–time profile of abiraterone in the dose-proportionality study

Following the administration of AA, the mean abiraterone plasma concentrations increased relatively rapidly to peak and declined in a biphasic manner, in both studies. The mean concentration–time profiles of abiraterone obtained in two studies are presented in Fig. 1.

### Dose-escalation study

The abiraterone plasma concentrations and exposures generally increased with increase in dose (Table 2). The inter-subject variability [coefficient of variation (CV %)] for *C*_max_, AUC_0–∞_, and AUC_0–*t*_ ranged from 38.8 to 53.6 % for the 250 mg/day cohort, 46.9–60.0 % for the 500 mg/day cohort, 57.0–58.4 % for the 750 mg/day cohort, and 32.7–40.8 % for the 1,000 mg/day cohort. The mean *t*_1/2_ ranged from 11.1 to 14.7 h in Cohorts 1 through 4. Abiraterone mean *t*_1/2_ and median *t*_max_ values appeared to be independent of dose.

**Table 2:** Pharmacokinetic parameters of abiraterone in dose-escalation study and dose-proportionality study (pharmacokinetic analysis set)

Parameter	Dose cohort 1 (250 mg/day) (*n* = 8)	Dose cohort 2 (500 mg/day) (*n* = 8)	Dose cohort 3 (750 mg/day) (*n* = 8)	Dose cohort 4 (1,000 mg/day) (*n* = 8)
*Dose*-*escalation study*				
*C*_max_ (ng/mL), mean (SD)	25.9 (10.0)	76.6 (35.9)	57.4 (32.7)	112 (36.7)
*t*_max_ (h), median (min, max)	1.75 (1.0, 4.0)	2.02 (1.0, 3.0)	1.75 (1.0, 3.0)	1.75 (1.0, 3.0)
AUC_0–last_ (ng.h/mL), mean (SD)	155 (83.3)	428 (257)	309 (180)	610 (249)
AUC_0**–∞**_ (ng.h/mL), mean (SD)	162 (84.7)	440 (254)	317 (183)	617 (249)
*t*_1/2_ (h), mean (SD)	11.1 (2.97)	14.6 (4.70)	14.7 (3.96)	12.7 (1.91)

	Dose cohort 1 (250 mg/day) (*N* = 27)	Dose cohort 2 (500 mg/day) (*N* = 29)	Dose cohort 3 (750 mg/day) (*N* = 28)	Dose cohort 4 (1,000 mg/day) (*N* = 29)
*Dose*-*proportionality study*				
*C*_max_ (ng/mL), mean (SD)	39.9 (25.3)	67.0 (34.7)	87.0 (43.3)	125 (76.4)
*t*_max_ (h), median (min, max)	2.0 (1.0, 6.0)	2..0 (1.0, 4.0)	2.0 (1.0, 4.0)	2.0 (1.0, 4.0)
AUC_0–last_ (ng.h/mL), mean (SD)	195 (109)	336 (156)	438 (189)	607 (298)
AUC_0–∞_ (ng.h/mL), mean (SD)	210 (105)	345 (155)	449 (189)	621 (300)
*t*_1/2_ (h), mean (SD)	14.4 (4.5)	15.3 (4.1)	16.5 (4.5)	16.0 (4.6)

*C*_max_ = the maximum plasma concentration; *t*_max_ = time to reach *C*_max_; AUC_0–last_ = area under the plasma concentration–time curve (AUC) from 0 to the last measurable plasma concentration; AUC_0–∞_ = AUC extrapolated to infinity

### Dose-proportionality study

Median *t*_max_ occurred at 2 h after administration and mean *t*_1/2_ ranged from 14.4 to 16.5 h. The mean exposures increased with increase in dose (Table 2). Inter-subject variability (CV %) was relatively high and ranged from 49.8 to 63.4 % for *C*_max_ and 42.0–55.8 % for the AUCs. Dose-normalized geometric mean ratios for *C*_max_ and AUC showed that abiraterone exposure decreased dose-proportionally at 750 mg (90 % confidence interval within 80–125 %) but not at 500 or 250 mg (Table 3).

**Table 3:** Dose-normalized pharmacokinetic parameters of abiraterone estimated in dose-proportionality study

Parameter	Dose of abiraterone acetate (mg/day)	Least squares means^a^	Ratios^b^	90 % confidence interval^c^
*C*_max_ (ng/mL)	250	117.97	124.06	102.94–149.52
	500	108.77	114.38	94.96–137.78
	750	98.00	103.06	85.52–124.21
	1,000	95.09		
AUC_0–last_ (ng h/mL)	250	630.29	120.56	103.63–140.26
	500	586.54	112.19	96.48–130.47
	750	523.43	100.12	86.06–116.48
	1,000	522.79		
AUC_0**–∞**_ (ng h/mL)	250	750.79	128.66	111.62–148.31
	500	663.45	113.69	98.71–130.96
	750	579.33	99.28	86.13–114.44
	1,000	583.54		

*C*_max_ = the maximum plasma concentration; AUC_0–last_ = area under the plasma concentration–time curve (AUC) from 0 to the last measurable plasma concentration; AUC_0–∞_ = AUC extrapolated to infinity

*PK* pharmacokinetic

^a^Least squares (LS) means based on dose-normalized pharmacokinetic parameters from the mixed-effects model, transformed back to the linear scale (i.e., geometric means)

^b^Ratio of parameter means (expressed as a percent), transformed back to the linear scale. Ratios are based on dose-normalized pharmacokinetic parameters

^c^90 % confidence interval for ratio of parameter means (expressed as a percent), transformed back to the linear scale. Confidence intervals are based on dose-normalized pharmacokinetic parameters

### Safety

In the dose-escalation study, overall, 23 TEAEs were reported by 9 (27.3 %) subjects: 2 each from 250, 500, and 1,000 mg/day cohorts and 3 from 750 mg/day cohort; all of the TEAEs were mild in nature. Overall, the most common TEAEs were diarrhea, nausea, vessel puncture site pain, dizziness, and rhinorrhea, each reported by 2 (6.1 %) subjects (Table 4). There were no discontinuations due to TEAEs. There were no serious adverse events (SAEs) or deaths reported during this study. No clinically significant changes were reported in hematological or clinical chemistry laboratory values, vital signs measurements, physical examinations, or 12-lead ECGs.

**Table 4:** Treatment-emergent adverse events (TEAEs) reported by ≥2 subjects in dose-escalation study and dose-proportionality study

*Dose*-*escalation study (N* = *33)*	
Number of subjects with at least 1 TEAE	9 (27.3 %)
Diarrhea	2 (6.1 %)
Nausea	2 (6.1 %)
Site conditions	3 (9.1 %)
Vessel puncture site pain	2 (6.1 %)
Eye disorders	2 (6.1 %)
Nervous system disorders	2 (6.1 %)
Dizziness	2 (6.1 %)
Renal and urinary disorders	2 (6.1 %)
Rhinorrhea	2 (6.1 %)
*Dose*-*proportionality study (N* = *32)*	
Number of subjects with at least 1 TEAE	10 (31.3 %)
Headache	2 (6.3 %)
Diarrhea	2 (6.3 %)
Vessel puncture site pain	2 (6.3 %)
Pain in extremity	2 (6.3 %)
Psychiatric disorders	2 (6.3 %)
Libido decreased	2 (6.3 %)
Erectile dysfunction	2 (6.3 %)

In the dose-proportionality study, overall, 10 (31.3 %) subjects [250 mg/day: 2 (7 %); 500 mg/day: 5 (17 %); 750 mg/day: 4 (14 %); and 1,000 mg/day: 3 (10 %)] experienced TEAEs. Overall, the most common TEAEs were headache, diarrhea, vessel puncture site pain, extremity pain, decreased libido, and erectile dysfunction, each reported by 2 (6.3 %) subjects (Table 4). None of the TEAEs led to discontinuations during this study. No SAEs or deaths were reported. There were no reports of TEAEs related to increased mineralocorticoid levels. No clinically significant changes were observed in hematological or clinical chemistry laboratory values (except that one participant showed grade 2 abnormality: high bilirubin level on day 17, which normalized on day 21), vital signs measurements, physical examinations, or 12-lead ECGs. Administration of AA resulted in decrease in serum testosterone levels and increase in serum LH levels; however, none of these changes were clinically significant.

**Fig. 2:**
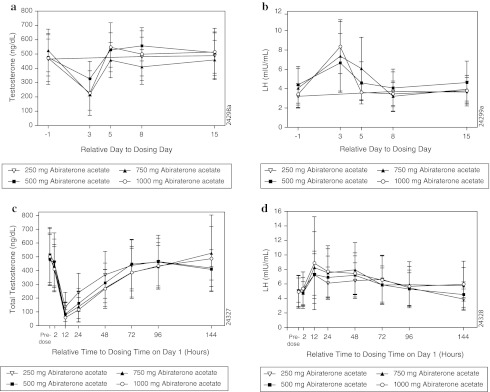
**a** Mean (SD) serum testosterone levels over time in dose-escalation study. **b** Mean (SD) serum luteinizing hormone levels over time in dose-escalation study. **c** Mean (SD) serum testosterone levels over time in dose-proportionality study. **d** Mean (SD) serum luteinizing hormone levels over time in dose-proportionality study. *n* = 8 for all treatment groups except for 500 mg abiraterone acetate group (*n* = 9)

In the dose-escalation study, mean serum total testosterone values were generally lowest on day 3 (the first postdose timepoint at which the testosterone levels were measured in the dose-escalation study) and approached the baseline values by day 5 (Fig. 2a), and mean serum LH values were highest on day 3 and approached the baseline values by day 8 (Fig. 2b). In the dose-proportionality study, oral administration of AA 250–1,000 mg/day resulted in transient decrease in the mean serum testosterone levels. The maximum decrease was observed at 12 h postdose (the first postdose timepoint at which the testosterone levels were measured in the dose-proportionality study), with mean values returning to baseline by 72 h for the 250 and 500 mg doses and by 144 h for the 750 and 1,000 mg doses (Fig. 2c). Administration of AA also resulted in increase in mean serum LH levels, with maximum increase observed at 12 h postdose (Fig. 2d).

## Discussion

Abiraterone acetate appeared to be fully converted to the active moiety abiraterone, as the majority of plasma concentrations for AA were below the limit of quantification in both the studies. There was a dose-related increase in exposure of abiraterone at doses from 250 to 1,000 mg in the dose-escalation study as well as in the dose-proportionality study. However, the dose-escalation study was not formally designed to assess the dose-proportionality. In the subsequent dose-proportionality study, statistical analysis of dose-normalized geometric mean showed that the decrease in abiraterone exposure from 1,000 to 750 mg was dose proportional, but the decrease at 500 and 250 mg was more than dose proportional (90 % CIs outside the 80–125 % range). In both studies, the mean *t*_1/2_ and median *t*_max_ values for abiraterone were similar across the evaluated dose range.

Total testosterone and LH levels measured in both studies indicated that the observed reduction in serum testosterone and elevation in serum LH following single dose oral administration of AA were reversible. In the dose-proportionality study, the maximum effect of AA on mean serum total testosterone and LH levels occurred at the 12-h postdose, which was the first postdose timepoint at which the testosterone levels were measured in this study. Visual inspection of the concentration–time profiles for abiraterone versus total testosterone and LH levels indicated an indirect pharmacokinetic/pharmacodynamic effect as shown by the delayed pharmacodynamic response.

In both studies, AA was safe and well tolerated in fasting healthy men, over the evaluated dose range. The most common TEAEs reported by at least 2 (6.1 %) subjects were diarrhea, nausea, vessel puncture site pain, dizziness, and rhinorrhea in the dose-escalation study and headache, diarrhea, vessel puncture site pain, extremity pain, decreased libido, and erectile dysfunction in the dose-proportionality study. There were no deaths, SAEs, or AEs, leading to discontinuation. There were no reports of adverse events of interest related to mineralocorticoid excess (e.g., fluid retention/edema, hypertension, and hypokalemia) and no adverse events of interest related to liver function test abnormalities or cardiac disorders.

Abiraterone acetate was found to be safe and well tolerated in previous phase I and II studies in patients with CRPC, when evaluated at doses of 250–1,000 mg/day; also, no dose-limiting toxicity was observed up to dose of 1,000 mg/day [12, 14–16]. The safety profile of AA observed in the present two studies was consistent with these studies and the other studies carried out in healthy men [17].

## Conclusion

Abiraterone acetate (250–1,000 mg/day) appeared to be rapidly converted to abiraterone, following single dose, oral administration in healthy men. Systemic exposure to abiraterone increased with increasing doses of AA. In the dose-proportionality study, abiraterone exposure was dose proportional between 750 and 1,000 mg doses; however, the exposure was slightly greater than dose proportional when exposures at 500 and 250 mg doses were compared with the exposure at the reference dose, 1,000 mg. Abiraterone acetate was generally well tolerated in healthy men, and the safety profile was consistent with the known toxicities of AA observed in other clinical studies in patients and in healthy men.
